# Antefebrin

**Published:** 1889-11-09

**Authors:** 


### ANTIFEBRIN.

Antifebrin and other allied bodies, being less soluble than antipyrin, are apt to manifest their cumulative effects sooner, and individual intolerance of these is more frequently observed. Thus, Dr. Khakanof, of Sophia, reports a case of a lady who, to relieve menstrual pain, took a 15-grain dose of antifebrin at bedtime and another next morning. She soon felt prostrated, became cyanotic, with irregular action of the heart, dyspnœa, thready pulse, convulsive twitchings of the face and limbs, and fall of temperature (96 F.). Dr. K., who was summoned immediately, tried various stimulants, hot bottles, etc., but without marked result. In the evening, after profuse diuresis, she recovered, slept well, and awoke next morning in her usual good health. Dr. Khakanof remarks that this lady had frequently taken fifteen-grain doses of antipyrin for the same purpose, with no unpleasant effects; that he has seen diuresis in some patients, and diaphoresis in others after antifebrin, but not both, and that while it is far from being devoid of danger, its physiological action is ill understood, and no antidote, properly so called, is known. Dr. Mollof, of the same town, has seen two cases of intense collapse, but without cyanosis, following eight-grain doses, though speedily yielding to stimulants.

